# What Shall We Treat?

**Published:** 1884-02

**Authors:** P. P. Wells

**Affiliations:** Brooklyn


### HOMŒOPATHIC PHYSICIAN,

A MONTHLY JOURNAL OF MEDICAL SCIENCE.

"If our school ever gives up the strict inductive method of Hahnemann, we are lost, and deserve only to be mentioned as a caricature in the history of medicine."—CONSTANTINE HERING.

Vol. IV. FEBRUARY, 1884. No. 2.

### WHAT SHALL WE TREAT?

In a report which has just come to my hand, of what appears as an abstract of the proceedings of the Pennsylvania State Homœopathic Medical Society at its late annual meeting, I find one of the members

" * * * expressed his doubts concerning the significance of the direction to treat patients and not the diseases. He always thought it was the physician's duty to treat the diseases and not the patients. It is our duty to direct our remedy at the unity of the group of symptoms. Each symptom of the case probably has the same central origin. We have to deal with symptoms as the outward expression of an inward disease. We ought to leave the patient, for the time being, out of sight."

The above is represented as from the author of the famous "last resolution" passed at the Indianapolis session of the American Institute of Homœopathy. It ought, perhaps, to surprise no one who has read that resolution and knows that the above utterances and that resolve have a common origin. It will at once be perceived by every one, even by those who are but slightly acquainted with the philosophy of Homœopathy, that if the practice of this member has been as far removed from this philosophy as are the above utterances, that he sorely needed the defense he was seeking in this resolution. If this practice has been so far from the requirements of the law of healing, which, by his position as member of the Institute and as editor of a periodical owned and conducted by a club of homœopathists, he may not unreasonably be supposed to have declared his confidence and belief in, it is quite conceivable that one who had so far departed from the truth he had been set to advocate and defend, was by an offended conscience impelled to seek support from some outside source, but certainly it is not a little singular that he should have had recourse to a body which was created for the defense and advancement of the truth he must have so grossly betrayed. It will be at once and readily seen that, such being his practice, he greatly needed defense from some quarter. Whether the one sought in this resolution can be of much avail to him may well be questioned.

If the utterances given above were from an old-school source they could cause no surprise. This school has always been "leaving" their "patients out of sight" and imagining a something distinct from them, which they have called diseases, and have been three thousand years floundering in their endeavors to grapple with that something therapeutically, which has till now eluded their grasp, and as a school, after these centuries, they stand, like Goethe's "Faust:"

> "Da steh ich nun, ich armer thor,
> Und bin so klug als wie zufore!"*

And yet this member of the Institute and editor affects to think this groping is the great thing to do; that, in short, the murk in which it must be pursued is to be preferred to the clear therapeutic light found, and only found, in the philosophy of the *Organon of Homœopathic Medicine.* And yet this man professes before the public to be a follower of Hahnemann and a believer and practitioner of the system of practical medicine he taught! And, still further and more strange, he does not appear to be in the least ashamed of this pretense.

He has "doubts concerning the significance of the direction to treat patients and not the diseases!" Why "doubts as to the significance"? There seems to be no necessary obscurity in the terms employed in this fundamental and most necessary rule, if one is to practice *homœopathic* medicine. There can be no reasonable doubts as to its "significance." Or, did the Doctor rather mean he had doubts as to the truth of the principle here inculcated? This may well be conceived of as his meaning, and the more, as, from the quotation at the head of this paper and his other utterances, we learn his innocence of all knowledge of homœopathic philosophy and his utter want of sympathy with its spirit

---

* "There stand I now, I, a poor fool,
As wise as I was before."

and teachings. What does the man mean* when he talks of treating diseases as something distinct from the patient? And distinct in such a degree and in such a way that in treating them the patient is to be "left out of sight!" He and all others may as well know that a knowledge of the symptoms is all the knowledge he or they have of any proper objective of treatment. That these symptoms, in their "totality," contain all that is or can be known of the modified life which we call sickness; that there can be no separation of these from the man who presents them; that they are his present modes of life and are no more separable from him than are any other processes of life function. To talk of diseases as something distinct from the patient or his symptoms is, perhaps, good old-school talk, but at this late day, when the expression of a better philosophy has been so long and so clearly before the world, is, for a professed homœopathist, no better than a talk to be ashamed of. Does this doctor mean by "treating diseases" treating the whole phenomena of the sick condition? We do not see what else he can mean. We reply, this is just what in homœopathic language is called the "*totality of the symptoms*," which alone constitute the basis of every true homœopathic prescription. And more than this, that this "totality" can no more be conceived of as a something distinct from the patient, than we can conceive of will-force independent of a living existence.

And then, the Doctor's idea of "the unity of the group of symptoms" is decidedly good. We do not remember to have seen the expression before. It is the more interesting coming from one who was supposed to have accepted the "totality of symptoms" as the only sure guide in therapeutic endeavors. What does he mean by this "unity," which seems, with him, so completely to have usurped the place given by homœopathic law to the "totality"? No wonder, after this, he needed the Indianapolis resolve.

"Each symptom in the case probably has the same central origin." What does he know about this and what if it has? Where has it, if it be so, the least practical relation to a true homœopathic prescription? This requires of us a simillimum to the "totality," and not in the least or at any time does a

* There would seem at times to be more than common difficulty in finding out just what this doctor does mean, by what he says, by the world at large. This will be apparent if it be remembered that his "resolve" was understood by all the world, besides himself, as an indorsement of any and every sort of a "go as you please" practice, while all the time, the doctor says, he only *meant* to refer to *consultations*. He ought to know what he meant. But if it were this it is certain nobody else did, till he told them.

"central origin" idea enter into it. It would be a great gain to our progress if those who set themselves up as teachers would remember that not all well-sounding sentences and phrases are freighted with common sense or truth. "Central origin" is good, but what is the meaning of it before the problem of a homœopathic cure. Does he mean by this "central origin" that there is, somewhere in the organism, a single point or part on which the morbid cause has made its impact, and that from this single part all symptoms of a sickness have their "origin"? If so, and we can see no other possible meaning, then we can only regret that before he uttered this opinion he had not discovered the fact that he knew just nothing at all of the matter of which he was speaking. It is not unlikely, even now, if he will give the idea a very little thought, he may discover his "probability" of this "central origin" only so much empty air.

There has seemed, more of late than before, to be prevalent a desire to get away from the duties and responsibilities of a practice under our law—something different, something better. The weakest exhibition of this that we have met is certainly this last notion of a possible homœopathic "treatment of *diseases* and not *patients*." P. P. WELLS.

